# Novel Surveillance Network for Norovirus Gastroenteritis Outbreaks, United States^1^

**DOI:** 10.3201/eid1708.101837

**Published:** 2011-08

**Authors:** Everardo Vega, Leslie Barclay, Nicole Gregoricus, Kara Williams, David Lee, Jan Vinjé

**Affiliations:** Author affiliations: Centers for Disease Control and Prevention, Atlanta, Georgia, USA (E. Vega, L. Barclay, N. Gregoricus, J. Vinjé);; Atlanta Research and Education Foundation, Decatur, Georgia, USA (K. Williams, D. Lee)

**Keywords:** norovirus, gastroenteritis, surveillance network, outbreaks, viruses, CaliciNet, United States, research

## Abstract

CaliciNet, the outbreak surveillance network for noroviruses in the United States, was launched in March 2009. As of January 2011, twenty state and local health laboratories had been certified to submit norovirus sequences and epidemiologic outbreak data to CaliciNet. During the network’s first year, 552 outbreaks were submitted to CaliciNet, of which 78 (14%) were associated with foodborne transmission. A total of 395 (72%) outbreaks were typed as GII.4, of which 298 (75%) belonged to a new variant, GII.4 New Orleans, which first emerged in October 2009. Analysis of the complete capsid and P2 region sequences confirmed that GII.4 New Orleans is distinct from previous GII.4 variants, including GII.4 Minerva (2006b).

## Abstract 2

TOC Summary: The launch of CaliciNet in March 2009 was a milestone.

Noroviruses are the primary cause of epidemic viral gastroenteritis and the leading cause of foodborne outbreaks in the United States (1–3). Although the course of disease is in most cases self-limiting, young, elderly, and immunocompromised persons are at risk for complications caused by severe vomiting and diarrhea (4–8). In addition to the clinical impact of norovirus disease, the economic effects in lost wages, time, and intervention procedures (e.g., clean-up costs and recalls) can be significant (9–11). Although norovirus outbreaks occur year-round, they are more common during the winter months (12–14).

Noroviruses are genetically classified into 5 genogroups, GI–GV, with GI and GII strains responsible for most human disease (2,15). GII viruses can be further divided into at least 19 genotypes, of which GII.4 is responsible for >85% of outbreaks (14,16), although other genotypes and viruses continue to circulate and cause sporadic disease in children (17–19). Over the past 15 years, new GII.4 variants have been identified; several have been associated with a global increase in the number of outbreaks (15). The last pandemic GII.4 variant, GII.4 2006b or GII.4 Minerva, was identified in late 2005/early 2006 and has been the predominant outbreak strain in the United States since then. The successive displacement of GII.4 variants suggests that population immunity is driving the evolution of GII.4 viruses (20,21), and the emergence of a new variant will cause an increase in the number of outbreaks in an immunologically naive population.

It is not fully understood why some GII.4 variants become pandemic whereas others do not. The combination of novel antigenic sites in protruding regions of the capsid (centered around amino acids 295 and 396) and the change or expansion of a susceptible population may be responsible for the emergence of pandemic variants (20,22). The latter theory has been supported by the discovery that different norovirus strains may have different histo–blood group antigen (HBGA) binding patterns and that nonsecretors are not susceptible to infection with certain genotypes or variants (23). Most mutations between genotypes and variants occur in the P2 region of the major capsid viral protein (VP), VP1, which contains the HBGA binding sites.

Since 2008, all 50 states have had the laboratory capacity for norovirus testing; the Centers for Disease Control and Prevention (CDC) National Calicivirus Laboratory (NCL) provides laboratory support to states that do not have in-house capacity for norovirus strain typing. Recent studies on the molecular epidemiology of norovirus in the US have been based on specimens from a subset of outbreaks that were submitted to CDC (13,24,25). To enhance and harmonize norovirus outbreak surveillance, CDC and its state partners have developed a national norovirus outbreak surveillance network, CaliciNet. CaliciNet was developed to improve standardized typing of norovirus outbreaks, assist in linking geographically different clusters of norovirus illness, allow rapid classification and identification of new norovirus strains, and establish a comprehensive strain surveillance network in the United States. In this article, we describe the CaliciNet network and report first-year results, including the identification of a new GII.4 norovirus variant.

## Materials and Methods

### CaliciNet

CaliciNet is a novel electronic laboratory surveillance network of local and state public health laboratories in the United States, coordinated by CDC. CaliciNet participants perform molecular typing of norovirus strains by using standardized laboratory protocols for reverse transcription PCR (RT-PCR) followed by DNA sequence analysis of the amplicons. A customized CaliciNet database developed in Bionumerics version 5.1 (Applied Maths, Austin, TX, USA) includes norovirus sequence and basic epidemiologic information (Table 1), which are submitted electronically via a secure connection to the CaliciNet server at CDC. Both epidemiologic and sequence data can then be used to help link multistate outbreaks to a common source (e.g., contaminated food). To ensure high-quality data entry, submissions to the CaliciNet server are performed by certified laboratory personnel of the participating state or local health laboratories, and final quality assurance/quality control is performed at CDC.

**Table 1:** Epidemiologic data fields required for upload to CaliciNet*

Required CaliciNet fields	Description
LabOBNumber	Year, outbreak, and number
Outbreak date	Begin date of outbreak
Outbreak city	City where outbreak occurred
Outbreak state	State where outbreak occurred
Outbreak setting	Select outbreak setting†
Outbreak country	Country where outbreak occurred
Transmission	Foodborne, person-to-person, waterborne
Conventional RT-PCR	Results of RT-PCR‡
Sequence experiment	Sequence of region D§

*RT-PCR, reverse transcription PCR. †Child care center, cruise ships, hospital, long-term care facility, party or event, restaurant, school and community, correctional center. ‡Region C or D. §Region D is the preferred sequence, but region C is also accepted.

CaliciNet certification for participants is a 2-step process that involves evaluation of data entry and analysis of sequences and a laboratory panel test. Each laboratory must pass an annual proficiency test. The laboratory certification and proficiency test consists of analyzing a panel of fecal samples by real-time RT-PCR and conventional RT-PCR analysis followed by bidirectional sequencing as described below. Certified participants are then authorized to upload norovirus outbreak data consisting of >2 samples per outbreak to the national CaliciNet database (Table 1). GII.4 sequences with >2% and 3% difference in region C or D, respectively, and >10% difference with all other noroviruses are further analyzed at CDC by amplification of the VP1 or P2 region.

### Outbreaks

All outbreaks submitted to CaliciNet and the NCL from October 2009 through March 2010 were genotyped by region D analysis (26). To verify GII.4 New Orleans variants, a subset of outbreaks from CaliciNet participating laboratories and 2 specimens from each outbreak received at the NCL from October 2009 through May 2010 were analyzed by using the P2 region as described below.

### Viral RNA Extraction

Viral RNA was extracted from clarified 10% fecal suspensions in phosphate-buffered saline with the MagMax-96 Viral RNA Isolation Kit (Ambion, Foster City, CA, USA) on an automated KingFisher magnetic particle processor (Thermo Fisher Scientific, Pittsburgh, PA, USA) according to the manufacturer’s instructions and eluted into 100 µL of elution buffer (10 mmol/L Tris pH 8.0 and 1 mmol/L EDTA). Extracted RNA was stored at –80°C until further use.

### Real-time RT-PCR

Viral RNA was tested for GI and GII noroviruses in a duplex format by using the AgPath-ID One-Step RT-PCR Kit (Applied Biosystems, Foster City, CA, USA) on a 7500 Realtime PCR platform (Applied Biosystems). The final reaction mix of 25 µL consisted of 400 nmol/L of each oligonucleotide primer, Cog1F, Cog1R, Cog2F, and Cog2R, and 200 nmol/L of each TaqMan Probe Ring 2 (27) and Ring 1C (28) (Table 2). Cycling conditions included reverse transcription for 10 min at 45°C and denaturation for 10 min at 95°C, followed by 40 cycles of 15 s at 95°C and 1 min at 60°C.

**Table 2:** Oligonucleotide primers and probes used for detection and genotype identification of norovirus strains submitted to CaliciNet*

Primer or probe name	RT-PCR target	Sequence, 5′ → 3′	Reference
TVN-L1	ORF2–ORF3	GGG TGT GTT GTG GTG TTG T_26_VN	(29)
L1	ORF2–ORF3	GGG TGT GTT GTG GTG TTG	This study
EVP2F	P2 (GII.4 specific)	GTR CCR CCH ACA GTT GAR TCA	This study
EVP2R	P2 (GII.4 specific)	CCG GGC ATA GTR GAY CTR AAG AA	This study
Cap D1	Region D GII	TGT CTR STC CCC CAG GAA TG	(26)
Cap C	Region D GII	CCT TYC CAK WTC CCA YGG	(26)
Cap D3	Region D GII	TGY CTY ITI CCH CAR GAA TGG	(26)
Cog 2F	ORF1–ORF2 junction (GII)	CAR GAR BCN ATG TTY AGR TGG ATG AG	(27)
Cog 2R	ORF1–ORF2 junction (GII)	TCG ACG CCA TCT TCA TTC ACA	(27)
Ring 2	ORF1–ORF2 junction (GII)	Cy5-TGG GAG GGC GAT CGC AAT CT-BHQ	(27)
Ring 1C	ORF1–ORF2 junction (GI)	FAM-AGA TYG CGI TCI CCT GTC CA-BHQ	(28)
Cog 1F	ORF1–ORF2 junction (GI)	CGY TGG ATG CGI TTY CAT GA	(27)
Cog 1R	ORF1–ORF2 junction (GI)	CTT AGA CGC CAT CAT CAT TYA C	(27)

*RT-PCR, reverse transcription PCR; ORF, open reading frame.

### Region D RT-PCR

The QIAGEN One-Step RT-PCR Kit (QIAGEN, Valencia, CA, USA) was used for region D amplification in a 25-µL reaction volume. RNAse Inhibitor (Applied Biosystems) was added to a final concentration of 15–20 units/reaction. Oligonucleotide primers CapD1, CapD3, and CapC were added to a final concentration of 1 µmol/L each (Table 2). RT-PCR conditions included reverse transcription at 42°C for 30 min and denaturation at 95°C for 15 min, followed by 40 cycles of 30 s at 94°C, 30 s at 40°C, and 30 s at 72°C. A final elongation step was run for 10 min at 72°C.

### P2 Region Amplification

The P2 region was amplified by using the SuperScript III One-Step RT-PCR with Platinum Taq High Fidelity Kit (Invitrogen, Carlsbad, CA, USA). The final reaction volume of 25 µL consisted of 4 µmol/L of EVP2F and EVP2R (Table 2). RT-PCR conditions included reverse transcription at 55°C for 30 min and denaturation at 94°C for 2 min, followed by 40 cycles of PCR at 94°C for 15 s, 55°C for 30 s, 68°C for 1 min, and a final extension step of 68°C for 5 min.

### Amplification and Cloning of GII.4 New Orleans

Novel GII.4 New Orleans sequences were identified by region D sequence analysis and further analyzed by amplification of complete open reading frame 2. Extracted RNA from fecal samples underwent cDNA synthesis with a TVN-L1 primer (29) (Table 2) for 60 min at 50°C by using the Superscript III cDNA synthesis kit (Invitrogen). The reaction mixture was purified by using the DNA Clean and Concentrator-5 (Zymo Research, Orange, CA, USA). The cDNA was amplified by using oligonucleotides (0.5 µmol/L each) L1 and Cog2F (Table 2), using the Phusion PCR Kit with the addition of 3% dimethyl sulfoxide (Finnzymes, Woburn, MA, USA). PCR conditions included denaturation at 98°C for 30 s followed by 40 cycles of 98°C for 10 s, 48°C for 30 s, and 72°C for 1.5 min. A final elongation step was run at 72°C for 10 min.

PCR products of ≈2.5 kb were gel purified and cloned by using a TOPO-TA Cloning Kit (Invitrogen). Five clones of each strain were fully sequenced bidirectionally and their respective consensus sequences were submitted to GenBank. The accession no. for GII.4 New Orleans is GU445325.

### DNA Sequencing

All amplicons were purified with the QIAquick Gel Extraction or PCR Purification Kits (QIAGEN) and sequenced by using the BigDye Terminator Kit version 1.1 (Applied Biosystems). Sequence reactions were cleaned up by using the BigDye Xterminator Kit (Applied Biosystems) and analyzed on a 3130XL Automated Sequencer (Applied Biosystems).

### Phylogenetic Analysis

VP1 or P2 sequences were aligned by using MEGA4 software (30). Maximum-likelihood phylogenetic analysis of VP1 amino acids were run in PhyML version 3.0 (www.atgc-montpellier.fr/phyml/binaries.php) by using the LG amino acids replacement matrix (31). The initial tree was the best of 5 random trees, and branches were supported by 100 bootstrap replicates. Branches with bootstrap support <60 were collapsed. The P2 sequence of representative GII.4 variants (GII.4 NOLA, GII.4 Osaka, GII.4 Yerseke [2006a], GII.4 Minerva [2006b], and GII.4 New Orleans) were also included in the analysis. Maximum-likelihood scores were generated for all models available in jModelTest by using the Akaike information criterion or the Bayesian information criterion (32) to select the best nucleotide replacement matrix for phylogenetic analysis. A transitional model with rate variation between sites, TIM3+G, had the best maximum-likelihood scores. The custom model was then run in PhyML version 3.0 with the approximate-likelihood ratio test calculated for branch support (33).

## Results

**Figure 1:**
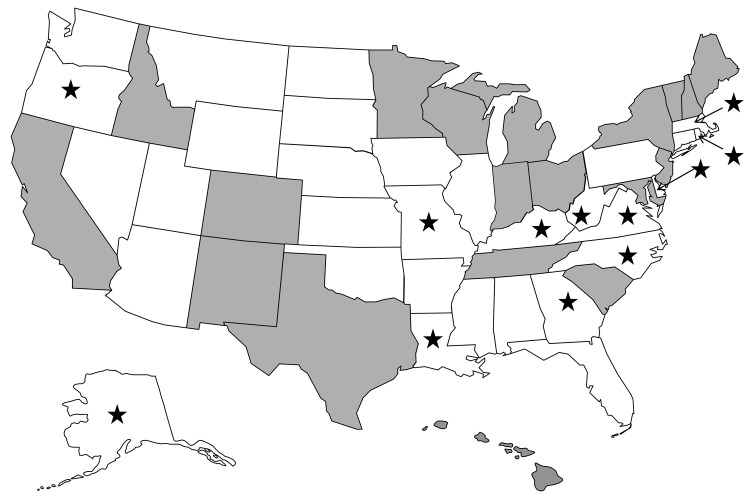
CaliciNet participating states (gray), nonparticipating states (white), and 12 states that submitted norovirus-positive specimens to Centers for Disease Control and Prevention for P2 analysis (stars).

As of February 2011, public health laboratories in 20 states have been CaliciNet certified (Figure 1); these states represent 53% of the US population (34). From the inception of CaliciNet in March 2009 through May 2010, 552 outbreaks were uploaded to the national CaliciNet database. Foodborne and person-to-person transmission were reported for 78 (14%) and 340 (62%) of the outbreaks, respectively, whereas the transmission route for the 134 remaining outbreaks was not reported.

**Figure 2:**
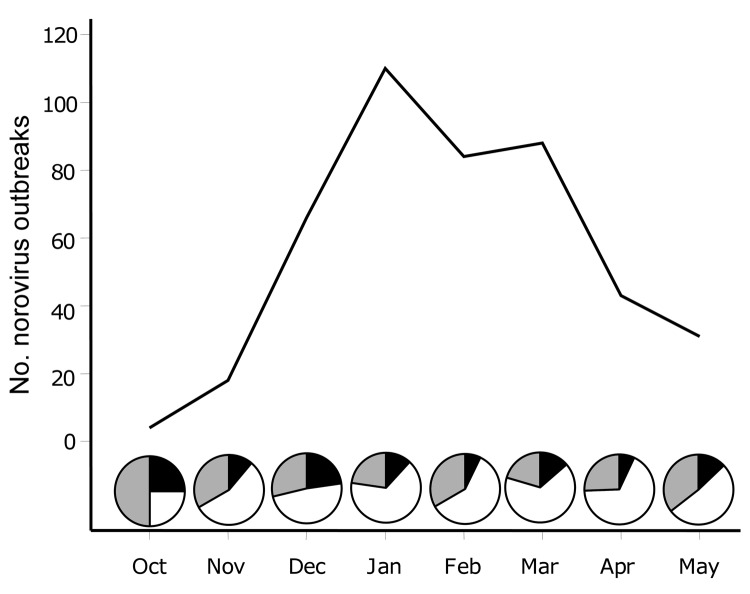
Gastroenteritis outbreak data submitted to CaliciNet from October 2009 through May 2010. Pie graphs represent the proportion of outbreaks reported as norovirus GII.4 New Orleans (white), norovirus GII.4 Minerva (black), and all other norovirus genotypes (gray).

GII.4 viruses caused 395 (73%) of the 552 outbreaks. The number of outbreaks increased from 4 in October 2009 to a peak of 110 in January 2010 and then decreased to 31 in May 2010 (Figure 2). A novel GII.4 variant (GII.4 New Orleans) was first identified in October 2009 and caused 56% of the outbreaks in November, compared with 11% caused by GII.4 Minerva. This novel variant remained the dominant strain in December 2009 and January 2010, causing 48% and 65% of the outbreaks, respectively. In February 2010, the number of outbreaks decreased to 84, but the proportion of GII.4 New Orleans outbreaks remained high (60%). In March 2010, GII.4 New Orleans accounted for 75% of the outbreaks. In April, total outbreaks decreased to 43, with 67% caused by GII.4 New Orleans and 7% by GII.4 Minerva; in May, total outbreaks were 31, with 52% caused by GII.4 New Orleans and 13% caused by GII.4 Minerva.

**Figure 3:**
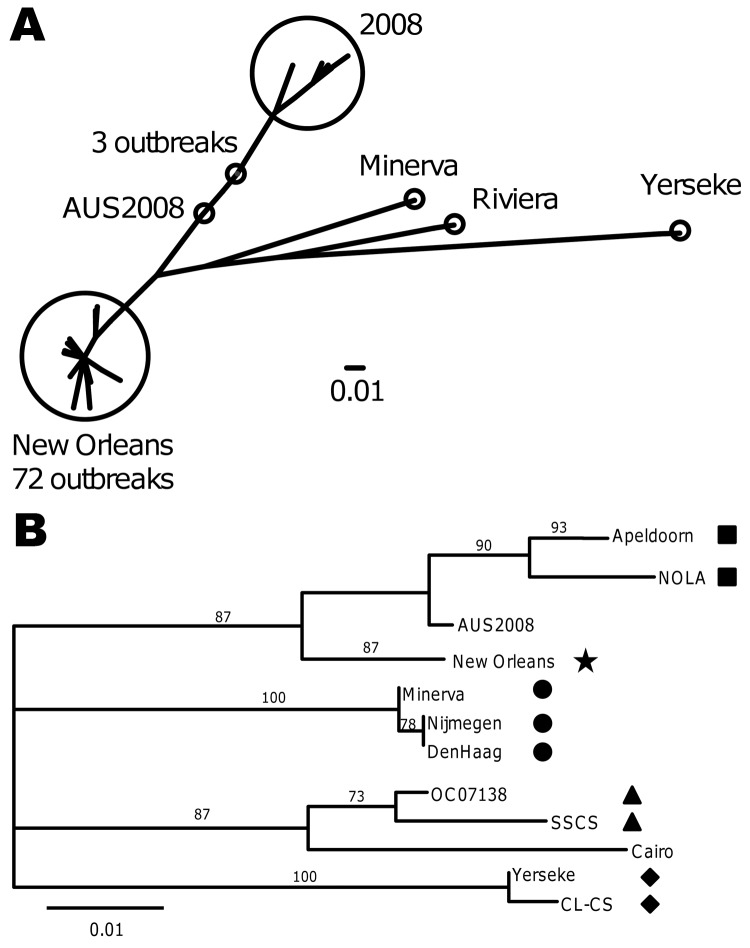
Unrooted phylogenetic tree of the P2 region from all norovirus GII.4 New Orleans strains submitted to CaliciNet and identified by region D analysis from October 2009 through May 2010 (A) and of the complete major capsid protein viral protein 1 of selected norovirus GII.4 variants (B). Numbers on branches represent bootstrap support out of 100. Symbols represent GII.4 variant types (nomenclature proposed by NoroNet in parentheses): black squares, GII.4 NOLA variant; star, GII.4 New Orleans (2010) variant; circles, GII.4 Minerva (2006b) variant; triangles, GII.4 Riviera (2007) variant; and diamonds, Yerseke (2006a) variant. AUS2008 (GenBank accession no. GQ845367) and Cairo (accession no. EU876888) are unidentified variant types. GenBank accession numbers of GII.4 sequences included in the analysis: Apeldoorn (AB445395), NOLA (GU270580), New Orleans (GU445325), Minerva (EU078417), Nijmegen (EF126966), DenHaag (EF126965), OC07138 (AB434770), SSCS (FJ411171), Yerseke (EF126963), and CL-CS (EU078419). Scale bars represent number of nucleotide (A) or amino acid (B) substitutions per site. P2 sequences can be provided upon request.

**Figure 4:**
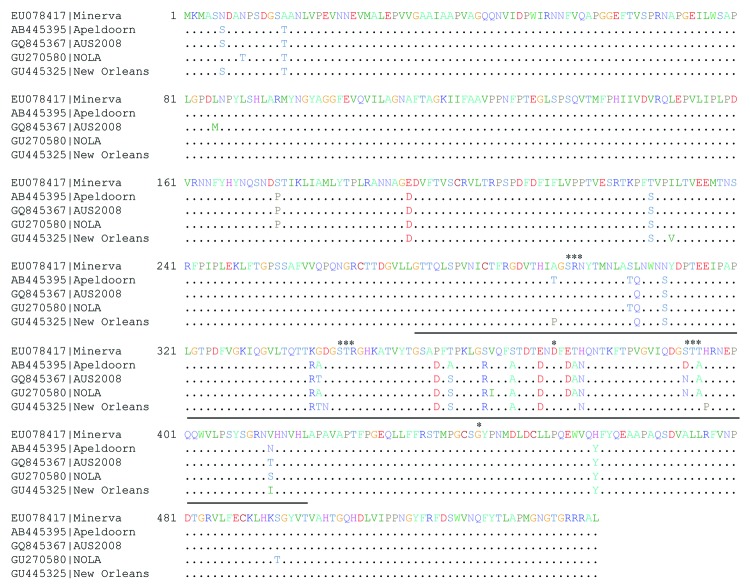
Amino acid substitutions in the major capsid protein viral protein 1 of norovirus New Orleans GII.4 strains compared with recent GII.4 variants. The P2 hypervariable region is underlined. *Protruding regions and histo–blood group antigen interacting sites. Dots indicate sequence identity.

Because region C and region D analyses are not able to differentiate genetically closely related GII.4 variants, we further analyzed the P2 region of 20 GII.4 New Orleans region D–positive strains from 4 CaliciNet states and sequenced the complete VP1 gene from a representative outbreak (Figure 3). Compared with recent GII.4 variants, GII.4 New Orleans had several amino acid substitutions, which were located near protruding regions (aa 294 and 396) and HBGA interaction sites (aa 339–341) (Figure 4).

In addition to the 20 strains from the CaliciNet-certified laboratories, the P2 region from 75 GII.4 outbreaks submitted by 12 non-CaliciNet states was sequenced (Figure 1). Of these, 72 (96%) outbreaks had P2 sequences with <2% nt difference compared with the prototype GII.4 New Orleans strain. Sequences from 3 outbreaks were closely related to a GII.4 variant first detected in Australia in 2008 (Figure 3).

## Discussion

The launch of CaliciNet in March 2009 was a milestone in the surveillance of norovirus gastroenteritis in the United States. CaliciNet enables standardized genotyping of norovirus strains, comparison of sequences from outbreaks that have a common source, and identification of new strains in real-time. The usefulness of CaliciNet was demonstrated during the winter of 2009–2010 when the emergence of a new GII.4 variant (GII.4 New Orleans) was identified. This new variant caused 60% of the outbreaks and replaced GII.4 Minerva as the predominant GII.4 strain. In addition, a new GII.12 strain caused 17% of the outbreaks during the winter of 2009–2010 (35).

GII.4 New Orleans was first detected in October 2009, and the proportion of all norovirus outbreaks it caused increased gradually to >50% during the winter months. Compared with known GII.4 viruses, GII.4 New Orleans had several changes in key amino acids in the P2 region of VP1 and around the sites that have been shown to be important in HBGA binding (20). Because most GII.4 variants that have been identified since 2004 are conserved at these sites, it has been speculated that mutations that change the HBGA binding pattern would decrease the fitness of the virus (36). During the last transitional period when GII.4 Minerva (GII.4 2006b) was identified, another GII.4 variant was co-circulating (21,37).

CaliciNet uses the same software as the US bacterial enteric pathogen surveillance network (PulseNet) (38), but it is customized with plug-ins to add CaliciNet-specific functionality. CaliciNet uses sequence data, whereas PulseNet is based on pulsed-field gel electrophoresis restriction digestion patterns of bacterial enteric pathogens. Current typing regions of CaliciNet target small regions of the norovirus genome, which makes it difficult to discern closely related norovirus strains, although the implications to human health may be significant. Our data and data from other studies (39) demonstrated that P2 region analysis enables more sensitive identification of new GII.4 variant strains compared with currently used CaliciNet regions. Use of these analyses would increase the sensitivity of outbreak surveillance to track strains that are part of a single outbreak and likely to have a common source. Hence, P2 is under consideration to be included in CaliciNet.

Like CaliciNet, the Foodborne Viruses in Europe network (FBVE) uses a central database to which users can submit norovirus sequences (40). Compared with the FBVE network, CaliciNet focuses primarily on noroviruses, is not web-based, and is based on a secured network connection to CaliciNet servers at CDC where the states log on as clients, enabling them to upload, view, and query outbreak data submitted by other states. CaliciNet also organizes training workshops and sends standardized protocols and annual proficiency panels to its members. The benefit of the FBVE network is that it can be more easily expanded to include laboratories outside its network, whereas to date CaliciNet allows only participants from state and local health laboratories in the US to participate.

The success of CaliciNet in linking multistate outbreaks to a common source (e.g., contaminated food) will depend on joint efforts of state and local epidemiologists to rapidly identify the likely common source and on CaliciNet laboratories for the timely upload of outbreak sequences to the national CaliciNet database. Although CaliciNet has selected region D as its preferred sequence region, a region C and soon a P2 region sequence database will be maintained to enable exchange of information with other norovirus surveillance networks. Because the region D assay targets a genetically highly heterogeneous region of VP1, the performance of this assay will be closely monitored over time, and necessary changes will be implemented to improve assay sensitivity and specificity. Future CaliciNet expansion will include other gastroenteritis viruses, such as sapovirus and astrovirus, as well as add capability for CaliciNet members to submit fecal samples from patients involved in norovirus-negative outbreaks to CDC for further testing, including novel pathogen discovery sequencing technologies (18).

CaliciNet was launched in March 2009 and helped in the rapid identification of a new GII.4 variant. P2 analysis confirmed that this variant was divergent from previous GII.4 viruses. The widespread presence of GII.4 New Orleans across the US coupled with the decreasing prevalence of the GII.4 Minerva variant, which has been the major cause of outbreaks during 2006–2009, suggests gradual strain displacement. Data from the 2009–2010 winter season showed the importance of CaliciNet and its future potential for norovirus surveillance in the US. To enhance norovirus surveillance globally, CaliciNet will collaborate with other norovirus surveillance networks, such as ViroNet in Canada and the global norovirus network, NoroNet (15), to better predict or determine norovirus epidemiologic or outbreak trends. International surveillance of viral foodborne outbreaks is essential because of the increasing globalization of the food industry.

Additional members of the Calicivirus network who contributed data (state represented): Chao-Yang Pan, Tasha Padilla (CA); Justin Nucci, Mary-Kate Cichon (CO); Gregory Hovan (DE); Precilia Calimlim, Cheryl-Lynn Daquip (HI); Edward Simpson (IN); Amanda Bruesch, Kari Getz (ID); Jonathan Johnston, Julie Haendiges (MD); Heather Grieser, John Martha (ME); Laura Mosher (MI); Elizabeth Cebelinski (MN); Alisha M. Nadeau, Fengxiang Gao (NH); Ondrea Shone (NJ); Frederick Gentry (NM); Gino Battaglioli (NY), Eric Brandt, Rebekah Carmen, Steven York (OH); Andrea Maloney (SC); Amy M. Woron, Christina Moore (TN); Chun Wang (TX); Valarie Devlin (VT); Tim Davis, Tonya Danz, and Jose Navidad (WI).
